# Promoting healthy aging through intergenerational exchange and digital empowerment: a pilot randomized controlled trial

**DOI:** 10.3389/fpsyt.2025.1637181

**Published:** 2025-07-16

**Authors:** Giulia Cossu, Stefano Lorrai, Alessia Galetti, Alessandra Perra, Massimo Tusconi, Giuseppe Carrà, Antonio Preti, Anita Holzinger, Mauro Giovanni Carta

**Affiliations:** ^1^ Department of Medical Sciences and Public Health, University of Cagliari, Cagliari, Italy; ^2^ University Hospital of Cagliari, Cagliari, Italy; ^3^ School of Medicine and Surgery, University of Milano-Bicocca, Monza, Italy; ^4^ Department of Neuroscience, University of Turin, Turin, Italy; ^5^ Teaching Center, Medical University of Vienna, Wien, Austria

**Keywords:** intergenerational programs, digital literacy, older adults, social isolation, social and biological rhythms

## Abstract

**Background:**

Social isolation and loneliness are major public health concern among individuals aged 65 and older, as they are associated with increased risks of morbidity and mortality. Intergenerational programs have emerged as promising interventions to mitigate these issues by fostering social participation and enhancing overall well-being. Interventions that also incorporate digital literacy support may further help address the relevant digital divide, which significantly contributes to social exclusion, particularly among older adults. This pilot study aimed to assess the feasibility and preliminary efficacy of a psychosocial intervention focused on intergenerational exchange and digital literacy.

**Methods:**

A 12-week crossover randomized controlled trial (RCT) design was employed. The intervention engaged younger and older participants in co-preparing seminar presentations, discussing these, and participating in plenary meetings—both in-person and online—on self-selected health, cultural, and topics meaningful from an individual perspective. Feasibility indicators included dropout rates and participant satisfaction. Preliminary measures of improvement were assessed using the Short Form Health Survey (SF-12) for quality of life, the Brief Social Rhythms Scale (BSRS) for the regularity of social and biological rhythms, and the Patient Health Questionnaire-9 (PHQ-9) for depressive symptoms.

**Results:**

A total of 12 participants were included in the experimental group and 9 in the control group. Feasibility outcomes showed an overall dropout rate of 28.57%, similarly to comparable trials. Notably, the attrition rate was lower in the experimental group (16.67%). Participant satisfaction was particularly high (M = 37.06, SD = 3.08). Preliminary analyses revealed a statistically significant improvement only in BSRS; 66.6% vs. 26.67%, *p* = 0.033. Trends toward improvement were observed in PHQ-9 and SF-12, although these did not reach statistical significance.

**Conclusions:**

The findings suggest very high satisfaction and moderate engagement among older adults involved in the program. Given the positive impact on the regularity of biological and behavioral rhythms—recognized as key protective factors in healthy aging—the improvement observed is particularly promising. Future studies with larger samples and extended follow-up periods are needed to corroborate this preliminary evidence.

**Clinical trial registration:**

ClinicalTrials.gov, identifier NCT06162871.

## Introduction

Social isolation, loneliness, and social disconnectedness are recognized as significant health risk factors for worsening quality of life and even premature mortality for individuals older than 65, comparable to smoking habits, obesity, and diabetes (1, 2). Health and social maintenance behaviors in older adults can be substantially disrupted by acute or chronic clinical conditions, frailty, or cognitive decline. These factors limit their capacity to actively engage in community life, thus increasing the risk of social disconnectedness (3, 4). Conversely, research highlights that older adults who maintain social connections experience lower rates of chronic and mental health conditions, including cardiovascular diseases and depression (5–7). Social identity and connectedness with family, friends, and the community are crucial for sustaining health, particularly when older adults face cognitive or physical impairments or crises like the recent COVID-19 pandemic (8–10).

The importance of addressing loneliness has been highlighted by the WHO as part of its active aging framework (12), which emphasizes the creation of supportive environments that promote social participation. In this context, the WHO advocates for the development of “age-friendly cities and communities” that enhance social capital and ensure equitable access to the social determinants of health and well-being, posing important challenges for aging policies within urban development (13–15).

Such an approach has provided valuable insights into the characteristics of age-friendly cities, leading to collaborative projects and initiatives that translate these principles into practice (16). Among these, intergenerational programs have proven effective in strengthening community connections and enhancing older adults’ health and well-being by promoting shared activities that generate mutual benefits (2, 17, 18). Designed to support sustained interactions between generations, these interventions foster competence, inclusion, and empowerment, while building meaningful intergenerational relationships. By reinforcing social cohesion and community bonds, intergenerational programs contribute to emotional, social, and physical well-being, underscoring their relevant role in promoting healthy aging (18), reduce social isolation, alleviate loneliness, and instill a sense of purpose among older adults (19).

Initiatives such as service-learning projects involving university students or family-based activities not only contribute to these aspects of well-being but also enable older adults to assume valued roles within their communities (20–22). Furthermore, evidence suggests that participation is associated with reduced psychological distress (23), improved memory function (24), and enhanced physical mobility (25).

The theoretical framework of intergenerational programs is based on Erikson’s lifespan development theory (26) and Allport’s contact theory (27). Erikson’s model emphasizes the mutual benefits of fostering relationships between children and older adults, highlighting how complementary developmental needs create unique synergies. In particular, after the age of 65, individuals reflect on their life achievements and seek meaning and coherence in their personal experiences. The need to maintain identity integrity and personal coherence is characteristic of later life; conversely, the loss of purpose and social roles may lead to feelings of regret, dissatisfaction, and emotional distress, increasing vulnerability to depression which is highly prevalent in older adults (5, 7). Intergenerational exchanges may serve as a valuable resource at this stage, offering older adults opportunities to transmit knowledge, share values, and assume meaningful social roles, thereby enhancing their sense of purpose and life satisfaction. Allport’s contact theory posits that interactions between distinct groups can reduce prejudice—such as ageism, which is a common form—and promote positive attitudinal change, offering valuable insights for the design and implementation of effective intergenerational programs (27). According to the theory, several key conditions must be met for contact to produce such outcomes: intergroup cooperation toward shared goals rather than competition; the presence of common objectives that foster a sense of unity and mutual interest; and institutional support, whereby social norms, policies, or organizational structures facilitate and legitimize the interaction. When applied to intergenerational interventions, this framework highlights the importance of designing activities that enable equal status exchanges, collaborative tasks, and jointly defined objectives between younger and older participants. However, the generational gap has been further amplified by the advent of digital technology and its integration into the routines of social interaction. Digital skills are typically more widespread among cohorts born in the digital era, while among older adults the digital divide has become a particularly significant factor contributing to social exclusion, especially during the COVID-19 pandemic. In Italy, for example, data from ISTAT (2019) indicate that only 34.0% of individuals aged over 65 have internet access. Younger, digitally proficient generations can therefore play a key role in facilitating exchanges with older, less technologically adept individuals. The positive psychosocial impact of internet use in people aged 65 and older has been well-documented, particularly in promoting social participation and alleviating depressive symptoms and feelings of loneliness (28, 29). Moreover, recent large-scale studies have further clarified the association between social isolation and dementia, identifying isolation as a significant risk factor, and emphasizing the potential of technology as a tool to foster social participation, especially in contexts where face-to-face interactions are limited, such as during the pandemic (30).

Given these premises, and considering that in many countries the growth of the average age of aging represents a major public health challenge (31), compounded by the scarcity of inclusion and active aging programs targeting health determinants in individuals over sixty-five, it becomes essential to develop interventions that demonstrate both feasibility and impact while addressing this complex scenario.

## Aim

This pilot study aims to assess the feasibility and preliminary improvements of a psychosocial intervention developed to promote social participation and active engagement through intergenerational exchange and digital support provided during the preparation and delivery of a series of mini-conferences. Strategically, these activities are expected to produce preliminary benefits in selected health-related outcomes.

In this work, in addition to the description of the intervention program across its phases, the main results related to feasibility (dropout rates and satisfaction) and preliminary measures of improvement induced by the intervention are presented about quality of life, depressive symptomatology, and the stability of biological and social rhythms.

## Methods

Design: This study was a12 weeks cross-over feasibility RCT design. Initially, the control group will remained inactive. Following the completion of the intervention by the experimental group, the control group subsequently participated in the same experimental intervention. As part of a complete cross-over design, the experimental group then served as the control group, given that the effects of the intervention do not persist over time (see **Supplementary Materials**).

The study received ethical approval from the Ethics Committee of the Azienda Ospedaliero-Universitaria di Cagliari, San Giovanni di Dio, Cagliari, under protocol number NP.2023/2534. All participants provided written informed consent prior to enrollment.

The trial was registered on *ClinicalTrials.gov* with the ID NCT06162871.

Timeline: The recruitment phase began in December 2023. Baseline assessments (T0) were conducted in January 2024. The intervention phase, including training and plenary sessions for the experimental group lasted 12 weeks. At post-intervention assessments (T1) the control group undergone the intervention, transitioning to the experimental phase as per the crossover design. The timeline can be summarized as follows: T0 (0 weeks); T1 (12 weeks); T2 (28 weeks)

Recruitment: During the recruitment phase, 106 individuals aged 65 years or older were contacted. These individuals had previously participated in studies on active aging conducted by the University of Cagliari and had provided consent to be re-contacted for further initiatives.

In line with previous research initiatives, participants residing in the metropolitan area of Cagliari, Italy, were therefore included.

Inclusion Criteria:

- Individuals aged 65 years or older
- Both sexes

Exclusion Criteria:

- Severe difficulties with independent walking
- Serious neurological conditions or disabilities preventing participation in the intervention either in person or remotely

Following consent to participate, Participants were randomized in a 1:1 ratio after the pre-assessment using computerized randomization.

Allocation was concealed using masked codes, and participants and outcome assessors were blinded to group assignment.

## Intervention

The intervention was entirely focused on empowering individuals over 65 years of age and highlighting their skills through exchanges with younger generations. All activities were conducted within the setting of a historic and monumental hospital located in the historic center of Cagliari, Italy. The initiative was hosted by the Azienda Ospedaliero Universitaria (AOU). Specifically, the hospital’s historic library and a conference hall were made available for the program.

The psychosocial intervention consisted of 12 weekly sessions, delivered both in-person and online.

Each session involved seminar-style activities, where participants over the age of 65 presented topics of their choice in rotation. These presentations were followed by discussions involving other participants with the presence of a moderator specialized in psychosocial rehabilitation during the presentations. The plenary discussion audience was composed of trainees from professional educator courses, young scholars from a cultural center, and its volunteers.

The sessions were designed to ignite and facilitate interactive discussions, with a strong emphasis on intergenerational exchange. The topics covered were cultural, historical, and related to well-being and health. Active participation was emphasized throughout all sessions to maximize engagement and collaboration.

The intervention aimed to highlight and utilize the expertise of participants aged over 65, allowing them to share their knowledge and experiences with others during the final presentations. Accordingly, the topics addressed during the sessions were aligned with the speakers’ areas of expertise. These included: gambling addiction, time management and lifestyle, history, traditions and folklore, migration, conflicts and wars, the relationship between humans and nature, classical historical literature, theater as a communication tool, the role of women, new technologies and AI, physical activity, and active citizenship.

All sessions were supported by project-provided facilitators and supervised by a psychotherapist specializing in active aging.

The entire intervention was structured within an intergenerational interaction framework, combining the preparatory phase — where younger professional educators provided digital and thematic support with continuous feedback to older adults in developing and rehearsing their presentations — with the plenary sessions, where trainees, young scholars, volunteers, and young professionals actively engaged in discussions, asked questions, and exchanged perspectives with older participants. This bidirectional dynamic fostered meaningful collaboration, reciprocal learning, and mutual competence development throughout the intervention.

The primary focus was to promote social inclusion, develop skills, enhance organizational abilities, and improve computer literacy among participants.

An additional component of the intervention involved providing support to individuals over 65 in developing digital and communication skills. This support was tailored to assist them in preparing their presentations and effectively using communication tools and the digital tools used to create the presentations, furthermore, providing useful digital skills when some of the participants opted to take part in the plenary sessions with online tool and needed to learn how to use them. This support was delivered by qualified professionals (professional educators) over the course of three sessions.

- First Session: Participants defined their chosen topic and outlined the steps for presenting it effectively.
- Second Session: The focus was on preparing the digital materials needed for their presentation.
- Third Session: Participants engaged in a full rehearsal of their presentation, simulating the actual delivery.

A fourth session was available for individuals requiring additional assistance. This included developing skills for using digital communication platforms (e.g., online platforms) or receiving further support to refine their presentations.

## Outcomes

### Intervention feasibility at post intervention time at T1 (12 weeks)

Feasibility is measured by the dropout rate, which is considered acceptable when around 20%-25% considering findings about dropout rates reported in the literature for randomized controlled trials (RCTs) involving old adults (32, 33).

### Intervention satisfaction at post intervention time at T1 (12 weeks)

To assess participant satisfaction, we used a self-report *ad hoc* questionnaire that was originally developed and applied by our research team in previous studies (34, 35). For the present study, the original version of the questionnaire was modified by adding two additional items specifically designed to capture the ecological validity and real-life applicability of the acquired skills, particularly considering that all activities in the present study took place within a hospital setting and our primary interest was to ensure that the skills acquired could be effectively transferred and applied outside of care environments. The final version consists of 8 items, each rated on a 5-point Likert scale, yielding a total score ranging from 8 to 40. The areas assessed include: overall satisfaction, perceived impact on general health, operator support, organizational support, fulfillment of expectations, willingness to recommend the intervention, perceived usefulness of the skills acquired, and frequency of skill utilization. The last two items specifically focus on participants’ perceptions regarding the applicability and transferability of the acquired skills to daily life—an aspect considered particularly relevant for this type of intervention. For the statistical analysis, mean scores and standard deviations (SD) were calculated based on the total score range.

### Preliminary improvement measures

#### Quality of life

To evaluate quality of life, the Short Form Health Survey (SF-12), a brief version of the SF-36 questionnaire, was used. It consists of twelve questions, with values ranging from 12 to 47, and includes the following dimensions: physical activity, disturbance in physical health, physical condition, self-assessment of health status, vitality, social activity, and mental health, assessed on a monthly basis. Higher scores indicate better quality of life. Its internal consistency is Cronbach’s α = 0.94 (36).

#### Regularity in biological and social rhythms

The Brief Social Rhythms Scale (BSRS), a 10-item questionnaire, with values ranging from 10 to 60, was used to assess the level of regularity in biological and social rhythms, specifically those related to sleep-wake cycles and appetite, as well as social contacts. Higher scores indicate worse regulation of rhythms. Cronbach’s α value is 0.912 (37).

#### Depression symptoms

The Patient Health Questionnaire-9 (PHQ-9) is a short self-administered tool, with values ranging from 0 to 27, used for screening symptoms of major depression according to DSM-IV over the last two weeks. It consists of 9 items, with higher scores identifying a greater presence of depressive symptoms. The internal consistency is Cronbach’s α = 0.89 (38).

### Statistical analysis

Descriptive analyses were conducted using percentages and frequencies for nominal variables, and means with standard deviations (M ± SD) for continuous variables. The normality of the main outcome variables at T1 in both the experimental group (EG) and control group (CG) was assessed using the Shapiro–Wilk test. Since the test did not support the assumption of a normal distribution, parametric tests were not applied. Instead, group comparisons were performed using Fisher’s Exact Test to evaluate the frequency of individuals showing improvement at T1 between the EG and CG. With regard to the preliminary assessment of intervention-induced improvements in the health-related variables of interest, operational evaluation will be based on the minimum percentage increase. All analyses were conducted using SPSS software (version 28.0.1.0; IBM, Armonk, NY, USA), and a two-tailed p-value < 0.05 was considered statistically significant.

## Result

A total of 12 participants were selected for the experimental group and 9 for the control group. Descriptive analyses reveal the main characteristic of the groups at the baseline time (**Table 1**)

**Table 1:** Characteristics of the sample at T0 experimental group (N=12) and control group (N=9).

Variables	Control group (n = 9)	Experimental group (n = 12)	Statistical test
Gender (Female)	4 (44.44%)	6 (50%**.)**	χ²(1) = 0.0636, *p* = 0.801
Age, mean ± SD	74.66±3.08 years	74.58± 2.74 years	*t*(19) = 0.0653, *p* = 0.9486
Education (Middle School; High School or more)	7 (77.78%)	12 (100%)	χ²(1) = 2.9474, *p* = 0.086

*χ²(df)*, chi-square test with degrees of freedom in parentheses. *t(df)*, Student’s t-test for independent samples with degrees of freedom in parentheses. SD, standard deviation. Percentages refer to the column total within each group.

### Feasibility

There were 2 dropouts in the experimental group (16.67%) and 4 in the control group (44.44%).

The reasons for dropout were related to personal commitments that prevented continued participation in the activities or health issues that arose during the trial. The average dropout rate between the two groups is 28.57%.

### Participant satisfaction

The overall mean score on the questionnaire was 37.06 (SD = 3.08), on a scale ranging from 8 to 40, reflecting a generally high degree of participant satisfaction.

### Measures of improvement on health outcomes

Comparisons between the experimental and control groups on the PHQ-9, BSRS, and SF-12 scores are presented in **Table 2**. The sample size reflects the crossover study design, in which the control group received the intervention after the experimental group completed it. Following a reasonable washout period, the experimental group subsequently served as the control group. Improvement rates were defined as positive changes in the total scores of the respective scales.

**Table 2:** Comparison between the experimental group and the control group on PHQ-9; BSRS; SF-12.

Outcomes	Experimental group N=15 t0	Experimental group N=15 t1	Control group N=15 t0	Control group N=15 t1	Fisher exact test	OR (CI 95%)
**PHQ-9** M±SD	3.33±1.71	3.33±1.71	4.20±3.36	4.2±3.36		
Normality Shapiro-Wilk Test p	0.2339	0.4302	0.2494	0.005		
Improvement t1		6 (40%)		3 (20%)	0.213	2.66 (0.52-13.65)
**BSRS** M±SD	19.46±7.29	19.73±5.49	17.66±5.09	20.40±5.77		
Normality Shapiro-Wilk Test p	0.399	0.660	0.253	0.697		
Improvement t1		10 (66.6%)		4 (26.67%)	**0.033**	5.50 (1.45-26.4)
**SF-12** M±SD	37.33±3.99	36.86±3.68	37.40±3.39	35.73±3.51		
Normality Shapiro-Wilk Test p	0.328	0.713	0.995	0.050		
Improvement t1		8 (53.3%)		5 (33.3%)	0.231	2.86 (0.52-10.01)
**Overall indicator** improvement considering PHQ-9, BSRS, and SF-12.		25 (53.33%)		12 (26.66%)	**0.009**	3.14 (1.30-7.59)

PHQ-9, The Patient Health Questionnaire; SF-12, Short Form Survey; BSRS, The Brief Social Rhythms Scale; M±SD, Mean, standard deviation; p, the p-value. Significant values are in bold.

Despite a trend toward improvement in the experimental group across all parameters, only BSRS shows a more pronounced improvement that reaches statistical significance (66.6% vs. 26.67%, Fisher’s Exact Test 0.033, OR 5.50, 95% CI 1.45–26.4), this corresponds to more than a fivefold increase in the likelihood of improvement in the experimental group.

Indeed, with regard to depressive symptoms the data highlight an improvement in favor of the experimental group (40% vs 20%); however, Fisher’s Exact Test did not reveal any statistical significance (p = 0.213, OR = 2.66, 95% CI: 0.52–13.65). Similarly, quality of life measured by the SF-12 indicated an improvement in the experimental group (53.3% vs 33.3%), although this difference did not reach statistical significance (p = 0.231, OR = 2.86, 95% CI: 0.52–10.01).

In contrast, when considering the overall improvements by summing the T0–T1 improvements of BSRS, PHQ-9, and SBRS, these are more frequent in the experimental group (53.3% vs. 26.67%, Fisher’s Exact Test 0.009, OR 3.14, 95% CI 1.30–7.59).

## Discussion

This pilot study aimed to explore the feasibility and preliminary effectiveness of an intergenerational psychosocial intervention designed to promote healthy aging through social participation. The results provide promising insights, however, the value for the mean between the two groups slightly exceeds the predefined standard of a maximum of 25% according to the predefined standard (32, 33). It should be noted, however, that in the experimental group, there were 2 dropouts, resulting in a much lower percentage (16.67%).

In contrast, the high dropout percentage in the control group (44.44%) may have been influenced, in the authors’ interpretation, by the expectation of being subsequently included in the experimental part of the trial, as per the crossover design. Over time, this expectation could have demotivated individuals from engaging fully in all phases while waiting to receive the intervention.

The program in all its phases, was generally well received, as reflected in the high average participant satisfaction.

Although not statistically significant, except for the domain of biological and social rhythm regulation, the observed changes may suggest a clinically relevant trend and warrant further exploration. In terms of health outcomes, indicators such as depressive symptoms and quality of life showed a tendency toward improvement in the experimental group; however, these improvements did not reach statistical significance.

However, a notable exception was found in the domain of biological and social rhythm regulation, as assessed by the BSRS. A significantly higher proportion of participants in the experimental group showed improvement compared to the control group, highlighting the potential benefits of a structured intergenerational program for older adults in re-establishing daily routines—an important factor for the psychological well-being of this population (37, 39).

However, it is important to consider that, although preliminary, these results carry relevance, as the regularity of behavioral and biological rhythms has been shown to be a fundamental protective factor even under exceptional stress conditions, such as those associated with the COVID-19 pandemic (9), and to play a protective role against the development of depression (10). It should be noted that social isolation—which is often a key component of rhythm irregularity—as well as the presence of depression in older adults, are among the main risk factors contributing to the development of conditions such as dementia and cardiovascular disease in this population (5–7, 39, 40). In addition, emerging evidence suggests that the dysregulation of behavioral and biological rhythms is a central component in the etiology of various disorders. In some cases, it may even be conceptualized as an autonomous syndrome when it presents as a state of hyperactivation or a vulnerable condition that can evolve into other disorders, depending on individual susceptibility and the specific nature or intensity of the stressor (41–43).

Additionally, when examining cumulative improvement across all primary outcome measures (BSRS, PHQ-9, and SF-12), the control group showed a significantly lower overall improvement rate. Although these outcomes are known to be closely interrelated, particularly in older adults, and the regularity of biological and social rhythms appears to play a crucial role, it is important to emphasize that these findings remain entirely preliminary. They are limited by the small sample size, as the current study represents a phase-two feasibility trial, and their clinical relevance should be interpreted as suggestive, warranting further investigation through larger studies, extended follow-up periods and in which these instruments can be applied with a more narrowly defined clinical focus. It should also be noted that perceived quality of life typically does not change drastically over a short period of weeks, and that this type of intervention should be framed within a preventive and health promotion perspective. Such interventions are expected to complement other health-oriented actions, potentially encouraging the adoption of healthier lifestyles and greater participation in community life. Similarly, chronic conditions such as depression in older adults are unlikely to show substantial improvement solely as a result of inclusion-based interventions, unless these are integrated as complementary strategies alongside appropriate pharmacological or clinical management.

Considering these aspects, it is important to highlight that the findings align with prior research suggesting that structured social participation and intergenerational interaction can positively contribute to various health outcomes (2, 16, 23).

The intervention, by focusing on enhancing the competencies and knowledge of older adults, is also consistent with the World Health Organization’s recommendations for age-friendly communities and active aging strategies (11, 12). Furthermore, the integration of digital training within the intervention—an area in which younger generations typically demonstrate greater proficiency—highlights a particularly promising approach to bridging the digital divide, which still constitutes a significant barrier to social inclusion among older populations, particularly, though not exclusively, in contexts such as Italy, where digital literacy among individuals over the age of 65 remains low (28, 30).

Regarding the selection of the characteristics of the intervention proposed in this study, a reflection can be made on the core components that constitute it. The fact that it appears to be a promising intervention is likely due to its adherence to several ideal characteristics, which have been shown to be more effective according to reviews and meta-analyses on interventions targeting social isolation in older people and on those implemented in community-residing older adults (2, 4, 16–18). Indeed the ideal intervention should meet the criteria of multidimensionality and integration, and promote active participation, socialization, and accessibility. An intervention that stimulates active engagement and a sense of personal value is crucial. Older adults must feel part of a meaningful project that fosters social inclusion and reduces loneliness. Socialization—both intergenerational and peer-based—plays a key role. The digital inclusion component represents an innovative aspect of this study. Although research in this area remains preliminary (28–30) it has shown promising results in improving well-being and reducing isolation (44, 45). When properly integrated, technology combined with social exchange and inclusion activities can help older adults remain connected to the world and enhance their independence. Strengthening digital literacy as a tool for empowerment is therefore a crucial element and alternating in-person and remote activities may serve as a strategic approach to facilitate a gradual familiarization with the technology considering that one of the main barriers for older adults in becoming familiar with technology is emotional in nature (45). However, it will be necessary to more carefully operationalize all the factors involved, starting from the assessment of digital competencies to the measurement of digital engagement. In future studies, this may include the use of validated instruments capable of capturing not only engagement but also confidence, satisfaction, and self-efficacy acquired through the intervention.

## Limitations

This study has several limitations that should be acknowledged.

- The small sample size limits the statistical power, and the cross-over design may have influenced the dropout rate observed, making it difficult to draw definitive conclusions about this data. Additionally, participant satisfaction is based only on those who completed the intervention in its entirety, meaning the results can be considered as a partial indication of the acceptability of this type of intervention.
- Furthermore, the data on improvement measures should be regarded as preliminary, not only due to the limited sample size but also because they are based on individuals who completed all phases of the study. Some characteristics of the participants who dropped out of the study may have influenced the results in a different manner. For example, the phenomenon of Volunteer Bias in Older Adults has already been described in studies focused on prevention, including elderly participants, but not exclusively. Volunteers for clinical trials often differ significantly from the general elderly population, tending to be healthier and more socially active (46). This bias can lead to an overestimation of the results, as these volunteers may not fully represent the broader group of older adults, particularly those with less active lifestyles. In addition, it should be acknowledged that a portion of the participants had previously been involved in other trials or structured activities, which may have further selected individuals already more engaged, motivated, and socially connected, potentially influencing both adherence to the intervention and its outcomes.
- Furthermore, the relatively short duration of follow-up limits the ability to assess the long-term effects of the intervention.
- A limitation of the present study is the absence of a direct measure of participants’ engagement, digital literacy, and of the competencies acquired through the intervention. Although some indirect indicators (e.g., BSRS scores, dropout rates, and participants’ satisfaction levels) provided partial insights about engagement, future studies with larger sample sizes and more targeted assessment tools will be necessary to explore these aspects in greater depth.
- Finally, although sex, age, and education level were balanced between the two groups at baseline, the small sample size did not allow for adjustment of the results for sociodemographic variables. This remains a relevant aspect that should be addressed in future studies with larger samples.

## Conclusion and future directions

With the global aging population steadily increasing, there is a critical need to design interventions that support health maintenance and ensure a high quality of life in later years. Although preliminary, the present findings suggest that intergenerational programs may be both feasible and beneficial in promoting psychological and social well-being in older adults. Given the importance of the regularity of biological and behavioral rhythms as one of the main protective factor for healthy aging—and considering the program’s socially engaging and intergenerational structure—such interventions deserve further development and evaluation in studies with larger sample sizes and extended follow-up periods to validate and expand upon these results. Furthermore, the integration of digital literacy and community-based approaches appears particularly promising for enhancing social inclusion and resilience among older adults.

## Data Availability

The data presented in this study are available upon request from the corresponding author. Due to privacy and ethical issues, data are not publicly available.
