# Supplementary figures and images for: Promoting healthy aging through intergenerational exchange and digital empowerment: a pilot randomized controlled trial
Source: Front Psychiatry. 2025 Jul 16;16:1637181. doi: 10.3389/fpsyt.2025.1637181 (PMC12308239; doi:10.3389/fpsyt.2025.1637181)

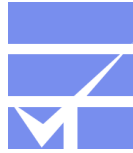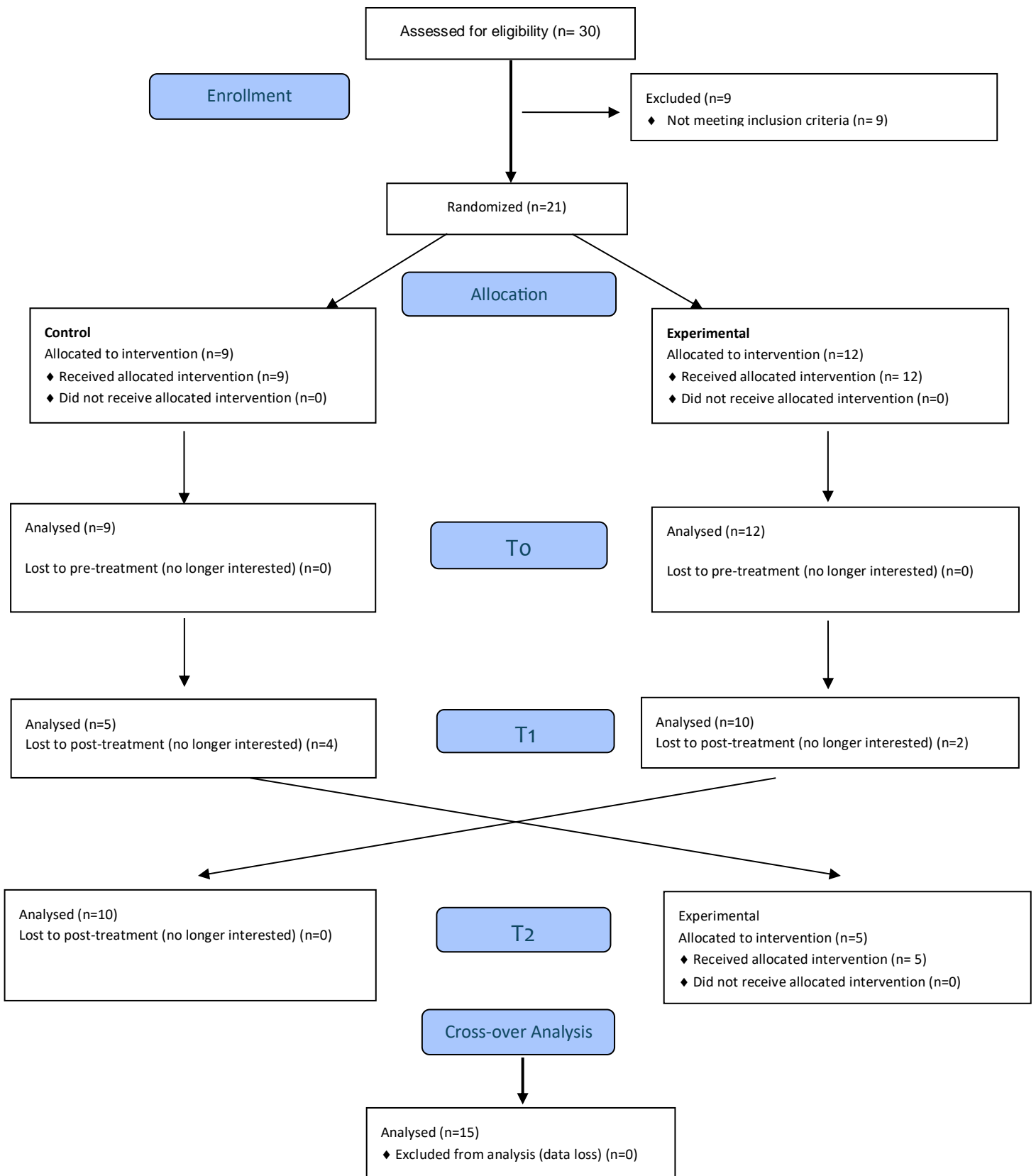

Supplement: Supplementary file 1 [file DataSheet1.pdf]
